# Cadmium-Tolerant and -Sensitive Cultivars Identified by Screening of *Medicago truncatula* Germplasm Display Contrasting Responses to Cadmium Stress

**DOI:** 10.3389/fpls.2021.595001

**Published:** 2021-03-11

**Authors:** Vanesa S. García de la Torre, Teodoro Coba de la Peña, José J. Pueyo, M. Mercedes Lucas

**Affiliations:** ^1^Instituto de Ciencias Agrarias, Consejo Superior de Investigaciones Cientificas, Madrid, Spain; ^2^Centro de Estudios Avanzados en Zonas Áridas, La Serena, Chile

**Keywords:** heavy metal stress, cadmium, reactive oxygen species, nutrient content, *Medicago truncatula*, germplasm, metal tolerance, NADPH recycling enzymes

## Abstract

Cadmium (Cd) pollution in soils is an increasing problem worldwide, and it affects crop production and safety. We identified Cd-tolerant and -sensitive cultivars by testing 258 accessions of *Medicago truncatula* at seedling stage, using the relative root growth (RRG) as an indicator of Cd tolerance. The factorial analysis (principal component analysis method) of the different growth parameters analyzed revealed a clear differentiation between accessions depending on the trait (tolerant or sensitive). We obtained a normalized index of Cd tolerance, which further supported the suitability of RRG to assess Cd tolerance at seedling stage. Cd and elements contents were analyzed, but no correlations with the tolerance trait were found. The responses to Cd stress of two accessions which had similar growth in the absence of Cd, different sensitivity to the metal but similar Cd accumulation capacity, were analyzed during germination, seedling stage, and in mature plants. The results showed that the Cd-tolerant accession (CdT) displayed a higher tolerance than the sensitive cultivar (CdS) in all the studied stages. The increased gene expression of the three main NADPH recycling enzymes in CdT might be key for this tolerance. In CdS, Cd stress produced strong expression of most of the genes that encode enzymes involved in glutathione and phytochelatin biosynthesis (*MtCYS*, *Mt*γ*ECS*, and *MtGSHS*), as well as *GR*, but it was not enough to avoid a redox status imbalance and oxidative damages. Our results on gene expression, enzyme activity, antioxidant content, and lipid peroxidation indicate different strategies to cope with Cd stress between CdS and CdT, and provide new insights on Cd tolerance and Cd toxicity mechanisms in *M. truncatula*.

## Introduction

Cadmium (Cd) is one of the most toxic substances for all living organisms, and its accumulation represents an increasing problem in agricultural soils due to anthropogenic activities, such as the extensive use of soil amendments and phytochemicals in agriculture, mining and industrial activities, transportation, uncontrolled dumping, and bad practices in waste treatment (Clemens et al., 2013). Plants are the main entry for Cd into the food chain (Peralta-Videa et al., 2009), presenting a risk both to the environment and to animal and human health. FAO is alerting of the hidden reality of soil pollution, especially in countries in development (Rodríguez-Eugenio et al., 2018). As an example, Cd levels in some areas of China have increased up to 250% in the last 30 years (Rodríguez-Eugenio et al., 2018), and in large cultivated areas, Cd accumulates to health-threatening levels in the so-called “cadmium rice” (Holdaway and Wang, 2018). Therefore, it is becoming of outmost interest the identification of crops and cultivars that are capable to tolerate moderate Cd stress and do not accumulate this heavy metal in their edible parts.

Legumes are together with cereals the main agricultural crops. They do not depend on nitrogen fertilization and are able to grow in poor and degraded soils, including heavy metal-polluted soils (Coba de la Peña and Pueyo, 2012). *Medicago truncatula* is a forage legume crop closely related to alfalfa, and a model legume plant. It constitutes an appropriate source for genetic improvement and an ideal model to study metal tolerance mechanisms in legumes. Cd-tolerant *M. truncatula* cultivars which do not accumulate Cd in the aerial part could be cultivated as a forage crop in moderately Cd-polluted soils, while cultivars with high metal accumulation could be used in phytoremediation of Cd-contaminated soils, due to its high biomass and good soil coverage. The identification of such cultivars in *M. truncatula* germplasm represents a potential tool to remediate the problems derived from Cd accumulation in both, arable lands and wild ecosystems. We have shown before the feasibility of identifying heavy metal-tolerant varieties in a *M. truncatula* germplasm by phenotyping a limited number of accessions (García de la Torre et al., 2013).

The effect of Cd stress on *M. truncatula* has been studied scarcely, mainly at the seedling stage (Aloui et al., 2009, 2011; Xu et al., 2010; Saeidi et al., 2012; Rahoui et al., 2014, 2015, 2017). Heavy metal tolerance and/or toxicity depend on the developmental stage of the plant, the metal concentration, and the time of exposure (Sanità di Toppi and Gabbrielli, 1999). In plants, Cd toxicity promotes growth reduction, macro and micronutrients uptake disturbance, and restriction in reserves mobilization at germination, among other detrimental effects (Benavides et al., 2005). Cd produces oxidative stress and lipid peroxidation (Romero-Puertas et al., 2012). In fact, the overproduction of reactive oxygen species (ROS) is considered the origin of the damage following Cd exposure (Cuypers et al., 2010). Cd does not take part in Fenton reactions for generating ROS but indirectly contributes to ROS formation by altering the antioxidant machinery of cells (Sharma and Dietz, 2009). Under Cd stress conditions, ROS play a dual role acting as toxic agents, but also as signal molecules, therefore the fine tuning of ROS homeostasis is crucial for the plant. The production of ROS and reactive nitrogen species with signaling functions are now considered strategies to avoid Cd toxicity (Romero-Puertas et al., 2019). Plants have several enzymatic and non-enzymatic antioxidant defense systems that allow ROS scavenging.

The first line of defense against ROS is the dismutation of O_2_^–^ to H_2_O_2_ through superoxide dismutase enzymes (SODs) and then catalases and peroxidases detoxify H_2_O_2_. There are also important antioxidant enzymes of the ascorbate-glutathione (ASC-GSH) cycle that allow maintaining the redox state of the cell. Some of these reactions are NADPH-dependent, and this molecule has been described as a limiting factor for the plant antioxidant capacity (Foyer and Noctor, 2011). NADPH is mainly produced by three enzymes, glucose 6-phosphate dehydrogenase (G6PDH), 6-phosphogluconate dehydrogenase (6PGDH), and NADP^+^-dependent isocitrate dehydrogenase (ICDH). Differential responses of these enzymes have been reported in stressed plants; and ICDH has been described as a key enzyme in NADPH recycling, and essential for the antioxidant defense under stress conditions (Marino et al., 2007). NADPH-recycling dehydrogenases are activated in response to Cd (Marino et al., 2013; Pérez-Chaca et al., 2014). There are several reports that study ROS production under Cd stress; however, most of them do not compare the differences between metal-tolerant and sensitive cultivars. Studies that analyze the antioxidant defense at different developmental stages are also scarce.

The aims of this work were to evaluate Cd tolerance in a *Medicago truncatula* germplasm, and to identify Cd-tolerant and Cd-sensitive genotypes. We also comparatively assessed Cd effects on tolerant and sensitive accessions at different developmental stages. Metal accumulation, nutritional status, and several markers for Cd tolerance, related with antioxidant defenses and Cd-induced oxidative stress, were analyzed. Our results suggest different strategies to cope with Cd stress depending on the Cd tolerance trait, and provide new insights on Cd response mechanisms in *M. truncatula*.

## Materials and Methods

### Plant Material

*Medicago truncatula* cv. Parabinga was used to set up the experimental conditions for the tolerance assays. Two hundred and fifty-eight *M. truncatula* accessions obtained from the National Plant Germplasm System of the United States Department of Agriculture (ARS-USDA) were used for the Cd-tolerance screening.

### Screening for Cadmium Tolerance

Seeds were scarified, sterilized, and germinated, and seedlings were transferred to a miniaturized hydroponic culture system containing modified Hoagland solution, pH 5.4, as previously described (García de la Torre et al., 2013). Seedlings were acclimatized during 24 h prior to Cd treatment.

*Medicago truncatula* cv. Parabinga seedlings were exposed to different Cd concentrations (0–40 μM CdCl_2_) for 48, 72, and 96 h under growth chamber conditions as described by García de la Torre et al. (2013). In subsequent experiments, seedlings of 258 *M. truncatula* accessions were exposed to 0 and 10 μM CdCl_2_ for 48 h. Thus, seedlings were 3 days old at harvest. Ten to thirty seedlings per cultivar were analyzed per treatment (control or Cd stress). Seedling relative root growth (RRG) was calculated as previously described (Sledge et al., 2005; García de la Torre et al., 2013), and it was used as an indicator of tolerance.

### Screening Validation in Growth Pouches

Two-day-old seedlings were acclimatized for 24 h prior to Cd exposure in growth pouches (CYG Seed Germination Pouches, Mega International, Minneapolis, MN, United States) containing 50 mL of nutrient solution. To set up the assay conditions, *M. truncatula* cv. Parabinga plantlets were grown in a range of Cd concentrations (0–200 μM CdCl_2_) for 12 days, under growth chamber conditions (García de la Torre et al., 2013). Nutrient solution was changed every 48 h. In subsequent experiments, seedlings of four potentially Cd-tolerant and four potentially Cd-sensitive accessions were exposed to 0 or 100 μM CdCl_2_ for 12 days. Thus, plants were 15 days old at harvest. Five to ten pouches, each containing five plants per cultivar and treatment, were analyzed. Root length, root and shoot fresh and dry weights, and number of leaves were measured, and relative parameters (RX) were calculated as follows: RX = (Parameter X_Cd_/Parameter X_C_) *×* 100.

### Cadmium and Nutrients Content

The content of Cd and nutrients was analyzed. At harvest, plants were dipped in 10 mM Na_2_EDTA, and then washed twice in ultrapure water. Roots and shoots were collected and stove-dried at 60°C for 3 days, and digested with nitric-perchloric acid (7:3). Contents of Cd and nutrients were determined using ICP-OES (Perkin-Elmer Optima 4300 DV). Three pouches, each containing five plants, per cultivar and treatment were analyzed.

### Analysis of Two Genotypes With Contrasting Tolerance

The tolerant PI 516929 (CdT) and sensitive PI 660497 (CdS) genotypes were selected to study the Cd effect on the germination process, gene expression, glutathione content, SOD and catalase (CAT) activities, and lipid peroxidation.

Scarified and sterilized CdT and CdS seeds were transferred into Petri dishes with a sterile filter paper imbibed with 5 mL of sterile water or 5 mL 200 μM CdCl_2_ for 72 h (25/19°C, 16/8 h) in the dark. Germination percentage was recorded on the third day. The seedlings radicles were removed and the cotyledons used in the α-amylase analysis. Four to six replicates (25 seeds per replicate) were assayed for each accession and treatment.

Three-day-old seedlings were transferred to the miniaturized hydroponic system for 72 h for acclimatization, prior to Cd treatment. Then, seedlings were exposed to 0 or 50 μM CdCl_2_ for 12 h. Five to ten seedlings per cultivar and treatment were used for histochemical detection of lipid peroxidation and loss of plasma membrane integrity. The rest of the roots were used for the gene expression analyses and determination of glutathione content.

Finally, 3-day-old CdT and CdS germinated seedlings were acclimatized for 7 days in vermiculite pots (350 mL), and then exposed to 0 or 100 μM CdCl_2_ for 15 days. Plants were 25 days old at harvest. Shoots and roots were analyzed for lipid peroxidation and CAT and SOD enzymatic activities. For each determination, four biological replicates per cultivar and treatment were analyzed.

### Alpha-Amylase Activity

Cotyledons were ground in liquid nitrogen and homogenized (0.1 g FW mL^–1^) in 2 mM imidazole-HCl buffer (pH 7.0). The homogenates were centrifuged (15,000 *g*, 1 h, at 0°C), and the supernatant was used to determine the α-amylase activity as described by Smith and Roe (1949). The reaction mixture (100 μL supernatant, 550 μL phosphate buffer, 0.9 μg μL^–1^ starch, and 0.5 M NaCl) was incubated during 30 min at 37°C, and the reaction was stopped with 80 μL of 1 N HCl. The remaining starch was stained with 700 μL of diluted lugol (1:6). A blank for each sample was prepared using 100 μL of supernatant, 650 μL of deionized water, and 700 μL of diluted lugol. The starch content was monitored at 620 nm. The α-amylase activity was expressed as μg hydrolyzed starch g^–1^ fresh weight min^–1^.

### RNA Isolation and Quantitative Real-Time PCR Analysis

Total RNA was isolated from seedling roots using the TRIZol (Invitrogen) method and treated with RNase-free DNase I (Thermo Fisher Scientific). RNA concentration was determined with a Nanodrop ND1000 spectrophotometer (Thermo Fisher Scientific). One microgram of DNA-free RNA was used for reverse transcription, using SuperScript^®^ II Reverse Transcriptase (Invitrogen), and oligo-d(T)18 as primer. The cDNAs were diluted 10-fold before performing qPCR. The Primer3 software^1^ was used to design specific primers for all genes tested (Supplementary Table 1).

Quantitative PCR reactions were run in a 7300 Real-Time PCR Sequence Detection System (PE Applied Biosystems). Each reaction contained 1 μL cDNA, 5 μL SYBR Green PCR master mix (PE Applied Biosystems), and 0.5 μM (final concentration) primer, in a total volume of 10 μL. Initial denaturing time (10 min, 95°C) was followed by 40 PCR cycles (95°C, 15 s; 60°C, 90 s; 72°C, 30 s), and a melting curve (95°C, 15 s; 60°C, 60 s; 95°C, 15 s). Relative gene expression was calculated according to the primer efficiency method (Pfaffl, 2001). Only fold-changes greater than two were considered as significant. Four biological replicates (10 roots each) per cultivar and treatment were analyzed.

### Glutathione Determination

Reduced glutathione (GSH) and total glutathione (GSH + GSSG) were measured by means of the glutathione recycling assay (Floreani et al., 1997), essentially as described by Shvaleva et al. (2010) with the following modifications: Roots were ground in liquid nitrogen and homogenized in 5% (w/v) sulfosalicylic acid (0.2 g FW mL^–1^). Homogenates were centrifuged (15,000 *g*, 5 min, 4°C), and the supernatant was used for determination of total glutathione. Reaction mixture (1 mL) included 25 μL of supernatant, 80 mM TEA (pH 8), 0.6 mM 5,5′-dithio-bis (2-nitrobenzoic acid) (DTNB, Sigma), 0.21 mM NADPH (Sigma), and 1 unit glutathione reductase (Roche, Branchburg, NJ, United States). DTNB, NADPH, and glutathione reductase were dissolved or diluted in 150 mM K-phosphate buffer (pH 7.4), 6.3 mM EDTA. The 2-nitro-5-thiobenzoic acid (TNB) generation was monitored spectrophotometrically at 412 nm for 1 min at 25°C. For GSSG determination, GSH was derivatized by adding 6 μL of 2-vinyl-pyridine and 10 μL 1 M triethanolamine (pH 8.0) to 90 μL of supernatant, for 30 min. The final pH of the reaction was between 6.0 and 7.0. Derivatized samples were assayed as described above. GSH was determined as the difference between total glutathione and GSSG. Four biological replicates (10 roots each) per cultivar and treatment were analyzed.

### Histochemical Analyses

Lipid peroxidation and loss of plasma membrane integrity detection were performed on intact seedling roots as described previously (Yamamoto et al., 2001). For lipid peroxidation, roots were stained for 20 min with Schiff’s reagent, which detects aldehydes originated from lipid peroxides, and kept in a solution containing 0.5% (w/v) K_2_S_2_O_5_ in 0.1 N HCl to retain the color.

The loss of plasma membrane integrity was detected by staining the roots with Evans blue solution [0.025% (w/v) Evans blue in 100 μM CaCl_2_, pH 5.6] for 10 min and washed several times with 100 μM CaCl_2_ (pH 5.6) until the dye no longer eluted from the roots.

The stained roots were observed under a stereomicroscope (Carl Zeiss Stemi 2000C, Fisher Scientific) with an attached digital camera (Leica DFC 420C). Color density was measured using the ImageJ software^2^. Five to ten seedlings per cultivar and treatment were analyzed.

### Lipid Peroxidation

Assessment of malondialdehyde (MDA) was performed using the thiobarbituric acid method (Singh et al., 2007). Shoots and roots were ground in liquid nitrogen, homogenized in 1 mL of 0.1% TCA (0.1 g FW mL^–1^ for shoots and 0.25 g FW mL^–1^ for roots), and analyzed as described by Redondo et al. (2009). Four biological replicates per cultivar and treatment were analyzed.

### Superoxide Dismutase and Catalase Activities

Shoots and roots (0.04 and 0.1 g FW mL^–1^, respectively) were ground in liquid nitrogen and homogenized in 50 mM K-phosphate buffer (pH 7.8) containing 0.1 mM EDTA, 1% (w/v) PVP-10, and 0.1% (v/v) Triton X-100. After centrifugation (20,000 *g*, 4°C, 30 min), the supernatant was used for the spectrometric determination of SOD and CAT activities, as described by Redondo et al. (2009).

### Statistical Analyses and Tolerance Index

The statistical analyses were performed with the IBM SPSS Statistics 20 software (SPSS Inc., Chicago, IL, United States). The root lengths from the screening assay were analyzed by ANOVA (*p* < 0.05; *n* ≥ 10). Cultivars that did not show significant differences or displayed a significantly higher root growth under Cd treatment than under control conditions were considered potentially Cd-tolerant. A Pearson’s correlation analysis was performed comparing the results obtained here with those previously reported on mercury and aluminum tolerance for the same *M. truncatula* germplasm (Sledge et al., 2005; García de la Torre et al., 2013). The relative parameters, fresh weight root/shoot ratio, and elements content were analyzed by ANOVA (*p* < 0.05). Fisher LSD or Tukey HSD tests were applied for pair-wise comparison. Data were combined considering the different accessions as replicates of potentially tolerant or sensitive plants, and a factorial analysis (principal component analysis method, PCA) was conducted with the different relative parameters. The resulting components (PC1 and PC2) were plotted to check the associations. For each accession, a tolerance index was calculated and normalized (nTI) as described by García de la Torre et al. (2013). Germination percentage was analyzed by the Pearsons’s chi-squared test. The glutathione content, lipid peroxidation, α-amylase activity, and SOD and CAT activities were analyzed by ANOVA (*p* < 0.05), and the Tukey HSD test was applied for pair-wise comparisons.

## Results

### *Medicago truncatula* Germplasm Screening for Cd Tolerance

The appropriate Cd treatment (concentration and time) for the screening assays was determined based on the root growth response of *M. truncatula* cv. Parabinga, a Cd-sensitive cultivar (Supplementary Figure 1). The treatment with 10 μM CdCl_2_ for 48 h had a clear effect on this cultivar without completely arresting root growth (RRG ≈ 30%), and it was selected for *M. truncatula* germplasm screening.

**FIGURE 1:**
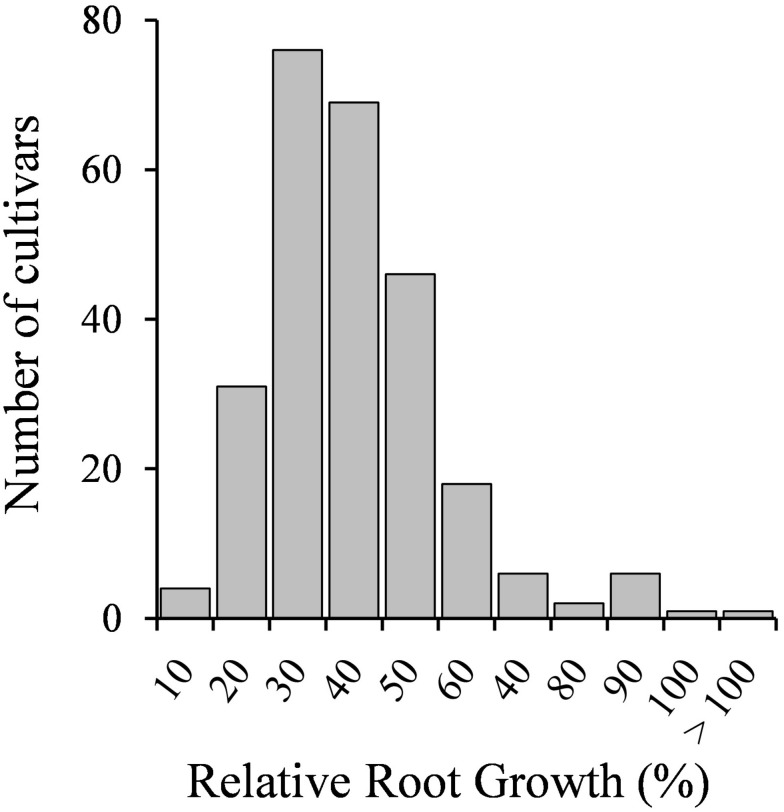
Seedling relative root growth (RRG) distribution of 258 *M. truncatula* accessions grown in the absence or presence of Cd stress (10 μM CdCl_2_, 48 h).

Seedlings of 258 *M. truncatula* accessions were tested for Cd tolerance. Root growth results are shown in Supplementary Table 2. The response to the metal of the different genotypes followed a normal distribution (Figure 1), suggesting that the number of tested accessions and the assay conditions were adequate. Most accessions displayed low RRG values, ranging from 10 to 50% (225 accessions), suggesting that *M. truncatula* is in general sensitive to Cd. Twenty-six accessions displayed values from 60 to 80%, and eight accessions exhibited RRG values above 80% (Supplementary Table 2). Cd had not a detrimental effect on the root growth of six of the accessions tested, as either there were not significant differences between control and Cd-treated seedlings, or the root growth values of Cd-treated seedlings were significantly higher than those of untreated seedlings (Supplementary Table 2).

**FIGURE 2:**
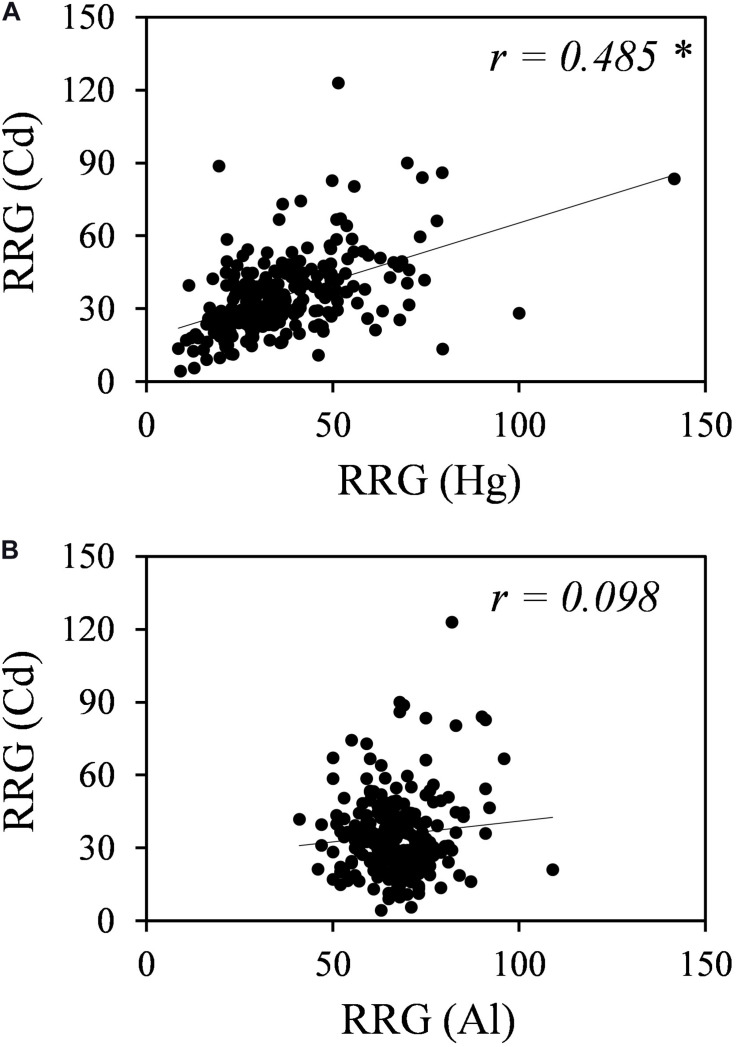
Correlation analyses for Cd and Hg tolerances **(A)** and for Cd and Al tolerances **(B)**. RRG values for Hg were obtained from García de la Torre et al. (2013), and RRG values for Al were obtained from Sledge et al. (2005). * Indicates significant correlation at *p*-value < 0.01.

Plants might have both metal-specific tolerance mechanisms and common mechanisms for different metals. A Pearson’s correlation analysis revealed no correlation between Al and Cd tolerances but a significant positive correlation between Cd and Hg tolerances (*r* = 0.486, *p* < 0.01) (Figure 2).

### Validation of the Rapid Screening Method for Cd Tolerance

To determine the validity of the screening results, treatment with 100 μM CdCl_2_ for 12 days in growth pouches was selected, as it was non-lethal but produced substantial differences in all growth parameters, in *M. truncatula* cv. Parabinga (data not shown).

Four potentially Cd-tolerant accessions (PI 660407, PI 660411, PI 516929, and PI 516933) with RRG values ranging from 66 to 90%, and four potentially Cd-sensitive accessions (PI 660497, PI 199257, PI 384634, and PI 516950) with RRG values ranging from 8 to 45% (Supplementary Table 2), were analyzed for morphological parameters. Results are shown in Table 1. No obvious phenotypic or anatomical differences were observed among accessions in the absence of Cd treatment. Root growth and root fresh weight were significantly affected in the presence of Cd in all accessions. While RRG and relative root fresh weight (RRFW) values of the potentially tolerant accessions were in general higher than those of the sensitive ones, two of the sensitive accession (PI 199257 and PI 660497) presented RRG values that were not significantly different from those of the tolerant accessions, and one tolerant accession (PI 516933) showed a similar RRFW value to those recorded for the sensitive varieties. No significant differences in relative root dry weight (RRDW) values were observed among varieties, and one sensitive accession (PI 660497) showed a significant reduction of the root dry weight after Cd treatment.

**TABLE 1:** Relative growth parameters and normalized tolerance indices for selected *M. truncatula* accessions grown in hydroponic pouches with 0 or 100 μM CdCl_2_ for 12 days.

Accession	RRG	RRFW	RRDW	RNL	RSFW	RSDW	R/S	nTI
**PI 660407**	41.46 ± 1.78^*a^	59.04 ± 6.00^*a^	91.95 ± 3.49^a^	100.00 ± 0.00^a^	100.36 ± 3.10^a^	94.89 ± 6.68^a^	73.94 ± 6.07^*a^	1.00
**PI 660411**	42.50 ± 0.89^*a^	46.60 ± 1.93^*abc^	88.87 ± 0.53^a^	100.00 ± 0.01^a^	77.99 ± 4.76^bc^	94.84 ± 6.57^a^	60.24 ± 3.80^*abc^	0.92
**PI 516929**	40.85 ± 1.59^*a^	52.61 ± 3.64^*ab^	83.38 ± 3.44^a^	93.47 ± 2.66^a^	87.73 ± 4.69^ab^	79.62 ± 3.71^ab^	62.80 ± 3.42^*abc^	0.90
**PI 199257**	35.11 ± 0.80^*ab^	42.06 ± 3.16^*bc^	86.83 ± 8.93^a^	64.58 ± 6.25^*b^	70.72 ± 7.38^*bcd^	79.83 ± 6.57^*ab^	53.24 ± 3.19^*bc^	0.78
**PI 516933**	40.34 ± 4.99^*a^	38.63 ± 5.31^*c^	74.94 ± 9.25^a^	85.71 ± 7.65^a^	60.26 ± 6.75^*d^	76.26 ± 8.55^ab^	68.06 ± 3.91^*ab^	0.75
**PI 516950**	29.26 ± 1.61^*b^	43.97 ± 6.21^*abc^	80.75 ± 14.57^a^	58.18 ± 8.39^*b^	72.00 ± 11.27^bcd^	76.96 ± 12.04^ab^	57.03 ± 5.15^*abc^	0.74
**PI 660497**	40.90 ± 1.77^*a^	44.33 ± 3.30^*bc^	72.70 ± 5.49^*a^	67.74 ± 1.97^*b^	64.22 ± 5.28^*cd^	66.75 ± 8.27^*b^	65.00 ± 3.45^*abc^	0.72
**PI 384634**	30.55 ± 1.42^*b^	33.64 ± 2.83^*c^	70.33 ± 8.59^a^	51.92 ± 5.76^*b^	67.30 ± 4.44^*cd^	79.83 ± 3.18^ab^	49.35 ± 2.52^*bc^	0.68

*RRG, relative root growth; RRFW, relative root fresh weight; RRDW, relative root dry weight; RNL, relative number of leaves; RSFW, relative shoot fresh weight; RSDW, relative shoot dry weight; R/S, fresh weight root/shoot ratio relative to control conditions; nTI, normalized Cd tolerance index. Mean values and standard errors are indicated. Asterisks indicate significant differences between control and Cd treatment for each accession (ANOVA, p < 0.05, n = 5–10). Different letters indicate significant differences between accessions for each relative parameter (LSD test, p ≤ 0.05).*

Regarding the Cd effect on the aerial part growth, the number of leaves decreased significantly in sensitive accessions following exposure to Cd, while non-significant differences were found for all potentially tolerant accessions. Tolerant accessions had relative number of leaves (RNL) values above 85%, and significantly higher than those of the sensitive accessions, which remained below 68%. Cd significantly affected the relative shoot fresh weight (RSFW) of accession PI 516933 (initially ranked as Cd-tolerant), while this parameter was not significantly affected for accession PI 516950 (initially ranked as Cd-sensitive). Only one potentially tolerant accession, PI 660407, presented an RSFW value that was significantly different from the potentially sensitive accessions. No significant differences were found between RSFWs for the potentially tolerant accessions PI 660407 and PI 5162929. Cd stress produced a significant decrease in the shoot dry weight of two sensitive accessions. Significant differences for relative shoot dry weight (RSDW) were found between the sensitive accession PI 660497 and the two tolerant accessions PI 660407 and PI 660411, which displayed the highest RSDW values. Cd significantly affected the root/shoot ratio (R/S) of all accessions, and accession PI 660407 showed the highest R/S ratio.

A PCA analysis revealed two components that accounted for 83.42% of the total variance. PC1 accounted for 49.02% of the explained variance, with the following eigenvalues:

$$\begin{array}{c} \mathrm{PC1}=\text{RNL}\times 0.46+\text{RRG}\times \left(-0.076\right)+\text{RRFW}\times \\ 0.568+\text{RRDW}\times 0.887+\text{RSFW}\times 0.875+\text{RSDW}\times 0.921 \end{array}$$

The second component accounted for 34.40% of the total variance, with the following eigenvalues:

$$\begin{array}{c} \mathrm{PC2}=\text{RNL}\times 0.749+\text{RRG}\times 0.959+\text{RRFW}\times 0.66+\\ \text{RRDW}\times 0.165+\text{RSFW}\times 0.347+\text{RSDW}\times 0.031 \end{array}$$

**FIGURE 3:**
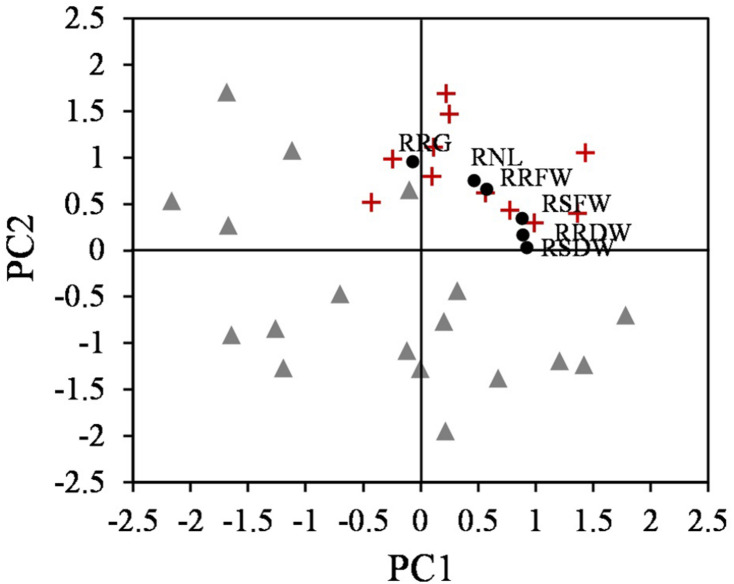
Scatter plot of the two components obtained from PCA that differentiate two groups, tolerant (+) and sensitive (Δ) accessions (*n* = 25), and the relative parameters according to the eigenvalues (∙).

Plotting the values for each component one against another, two different groups were identified, corresponding to tolerant and sensitive categories (Figure 3). These groups were associated with both components, and the tolerant accessions exhibited higher values than the sensitive accessions for the PCs. The RSDW and RRDW for PC1 and the RRG for PC2 were the main contributors to the maximum principal component variation. A normalized index of Cd tolerance (nTI), ranging from 0 to 1, was generated and a ranking of tolerance for the analyzed accessions was obtained (Table 1). The nTI values for the initially ranked as Cd-sensitive accessions were lower than 0.80. PI 660407 was the most Cd-tolerant among the accessions tested, and PI 516929 and PI 660411 could be confirmed as Cd-tolerant. The nTI values for those three tolerant accessions ranged from 1.00 to 0.90. Accession PI 516933, which was initially classified as Cd-tolerant according to the screening, presented an nTI value below 0.80, and therefore it had to be re-classified as a sensitive accession.

**FIGURE 4:**
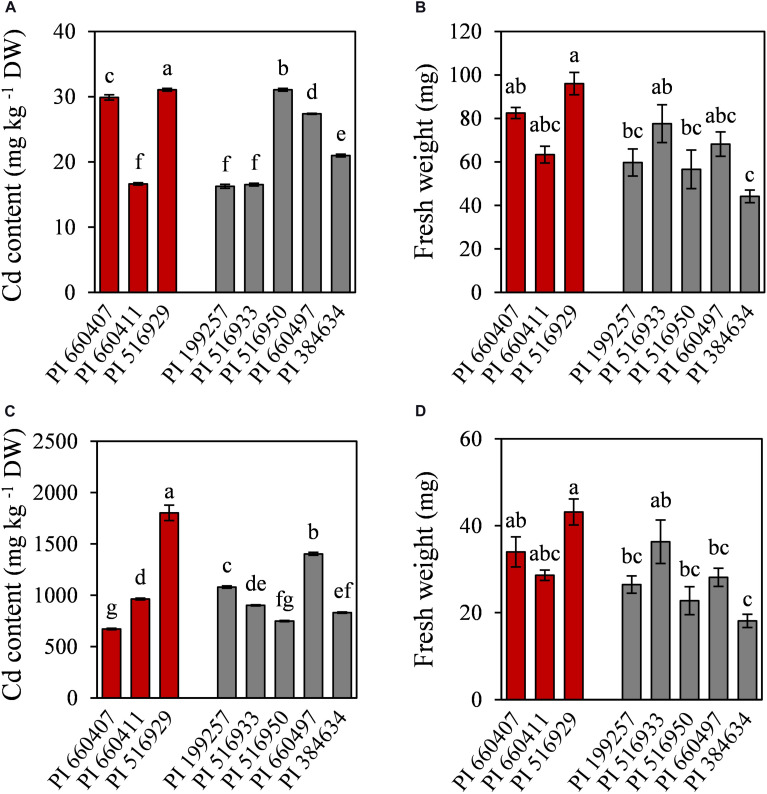
Cadmium content and fresh weight of shoots **(A,B)** and roots **(C,D)** of tolerant (red) and sensitive (gray) *M. truncatula* accessions grown in hydroponic pouches under Cd stress (100 μM CdCl_2_, 12 days). Mean values ± standard error are indicated, *n* = 3 for Cd content; *n* = 5–10 for fresh weight. Different letters indicate significant differences between cultivars (Tukey HSD test, *p* ≤ 0.05).

To further characterize the eight selected accessions, Cd accumulation and nutrients content were determined in roots and shoots. Cd was mainly accumulated in roots and only low amounts of Cd were translocated to the shoots (Figure 4). Cd-tolerant accession PI 516929 accumulated the highest amount of Cd in roots (1802 mg kg^–1^) and shoots (32 mg kg^–1^), while Cd-tolerant accession PI 660407 had the lowest Cd content in roots (672 mg kg^–1^) and high Cd content in shoots (29 mg kg^–1^). Among the Cd-sensitive accessions, the highest Cd content in roots was found in PI 660497, which also had high Cd content in shoots. The lowest concentration of Cd in roots was observed in PI 516950, whose shoots showed the highest Cd content. Cd content in roots and shoots did not appear to correlate with the tolerance trait (Cd-tolerant or Cd-sensitive) and therefore, Cd accumulation cannot be considered as a suitable predictor for Cd tolerance.

The Cd effect on the macro and micronutrients contents in roots and shoots of the different tested accessions was diverse (Tables 2, 3), but common features could be observed. In all accessions, Cd caused a significant decrease in Ca and S contents in shoots, and in Mn content in roots. The P content in roots and shoots was also decreased by Cd in all accessions, although it was not statistically significant for PI 660411 roots. Cd negatively affected the content of Mg in shoots of all accessions, although the decrease of Mg content was not statistically significant for PI 516929 and PI 384634. Following Cd stress, S content increased significantly in roots of tolerant accessions as well as in the sensitive accession PI 199257, and it decreased or did not change in the roots of the rest of the sensitive accessions. For the rest of nutrients, the effect of Cd was quite heterogeneous and did not appear to correlate with tolerance.

**TABLE 2:** Macronutrients content in Cd-tolerant and Cd-sensitive accessions of *M. truncatula* grown in hydroponic pouches with 0 or 100 μM CdCl_2_ for 12 days.

Accession		PI 660407		PI 660411		PI 516929		PI 199257	

Nutrient (μg g^–1^)	CdCl_2_ (μM)	Root	Shoot	Root	Shoot	Root	Shoot	Root	Shoot
**K**	0	48148	72238	59248	68454	40749	66033	50577	83970
	100	42685^∗^	44657^∗^	51069^∗^	60056^∗^	44605	71336	54814	60665^∗^

**Ca**	0	4174	3638	3095	3597	2648	4244	3637	4871
	100	5675^∗^	263^∗^	3013	1875^∗^	2442^∗^	2663^∗^	3243^∗^	2075^∗^

**Na**	0	1809	595	1487	545	1348	517	1643	675
	100	2420^∗^	505^∗^	843^∗^	517^∗^	938^∗^	500	978^∗^	5106^∗^

**Mg**	0	1399	1576	1036	1664	1206	1456	1211	2069
	100	1617^∗^	831^∗^	1083	1352^∗^	1075^∗^	1385	1228	1562^∗^

**P**	0	3089	5956	3890	4377	3454	3944	5793	5143
	100	2848^∗^	2230^∗^	3689	3170^∗^	2656^∗^	3004^∗^	5213^∗^	3516^∗^

**S**	0	1311	1963	880	1151	1757	2557	1174	1296
	100	2056^∗^	1138^∗^	1413^∗^	951^∗^	2330^∗^	1329^∗^	1546^∗^	1028^∗^

**Accession**		**PI 516933**		**PI 516950**		**PI 660497**		**PI 384634**	

**Nutrient (μg g^–1^)**	**CdCl_2_ (μM)**	**Root**	**Shoot**	**Root**	**Shoot**	**Root**	**Shoot**	**Root**	**Shoot**

**K**	0	58245	79092	51150	74378	50838	56325	44575	64135
	100	52258^∗^	71325	51131	56235^∗^	44686^∗^	53260	48576^∗^	53056^∗^

**Ca**	0	3375	5721	3191	3850	2517	4419	4123	2380
	100	3205^∗^	2596^∗^	3863^∗^	1336^∗^	3946^∗^	2145^∗^	2952^∗^	822^∗^

**Na**	0	1443	654	1535	610	1353	423	1983	597
	100	1106^∗^	525^∗^	1826^∗^	886^∗^	882^∗^	417	1650^∗^	562

**Mg**	0	1025	1672	1115	1757	1080	1697	1689	1347
	100	1204^∗^	1499^∗^	1484^∗^	1600^∗^	978^∗^	1552^∗^	1469^∗^	1278

**P**	0	3856	5007	4798	5329	5570	5375	3015	5576
	100	3545^∗^	4097^∗^	2740^∗^	3819^∗^	4608^∗^	4017^∗^	2835^∗^	3848^∗^

**S**	0	2246	1870	2656	2690	1702	2176	2199	2327
	100	1754^∗^	1163^∗^	2027	1471^∗^	1820	1036^∗^	1526^∗^	1393^∗^

*Asterisks indicate significant differences between treatments for each cultivar (ANOVA, LSD test, p ≤ 0.05, n = 3).*

**TABLE 3:** Micronutrients content in Cd-tolerant and Cd-sensitive accessions of *M. truncatula* grown in hydroponic pouches with 0 or 100 μM CdCl_2_ for 12 days.

Accession		PI 660407		PI 660411		PI 516929		PI 199257	

Nutrient (μg g^–1^)	CdCl_2_ (μM)	Root	Shoot	Root	Shoot	Root	Shoot	Root	Shoot
**Fe**	0	225	225	102	71	73	51	116	69
	100	353^∗^	80^∗^	110^∗^	55^∗^	108^∗^	52	80^∗^	68

**Mn**	0	209	55	127	40	239	41	142	49
	100	45^∗^	31^∗^	93^∗^	49^∗^	80^∗^	45^∗^	85^∗^	47

**Zn**	0	240	38	80	41	77	27	55	34
	100	110^∗^	67^∗^	46	47	62	30 *	70 *	24 *

**Cu**	0	206	27	106	35	38	14	57	31
	100	192^∗^	64^∗^	60^∗^	29^∗^	46^∗^	26^∗^	75^∗^	15^∗^

**Accession**		**PI 516933**		**PI 516950**		**PI 660497**		**PI 384634**	

**Nutrient (μg g^–1^)**	**CdCl_2_ (μM)**	**Root**	**Shoot**	**Root**	**Shoot**	**Root**	**Shoot**	**Root**	**Shoot**

**Fe**	0	141	55	126	75	121	57	290	88
	100	401^∗^	61^∗^	477^∗^	173^∗^	105^∗^	56^∗^	126^∗^	71^∗^

**Mn**	0	189	45	239	50	267	48	204	61
	100	96^∗^	48^∗^	28^∗^	61^∗^	69^∗^	40^∗^	52^∗^	42^∗^

**Zn**	0	104	44	181	38	133	40	164	140
	100	117^∗^	32^∗^	299^∗^	67^∗^	79^∗^	37^∗^	173^∗^	42^∗^

**Cu**	0	136	27	126	75	71	21	208	75
	100	108^∗^	17^∗^	477^∗^	173^∗^	84^∗^	25^∗^	165^∗^	23^∗^

*Asterisks indicate significant differences between treatments for each cultivar (ANOVA, LSD test, p ≤ 0.05, n = 3).*

### Contrasting Responses to Cd Stress of Two Accessions With Different Sensitivity to Cd

The responses to Cd stress were analyzed in two accessions classified as Cd-tolerant (CdT; PI 516929) and Cd-sensitive (CdS; PI 660497), respectively. Both accessions were similar in size in the absence of Cd and presented similar accumulation of Cd in roots and in shoots following Cd exposure.

**FIGURE 5:**
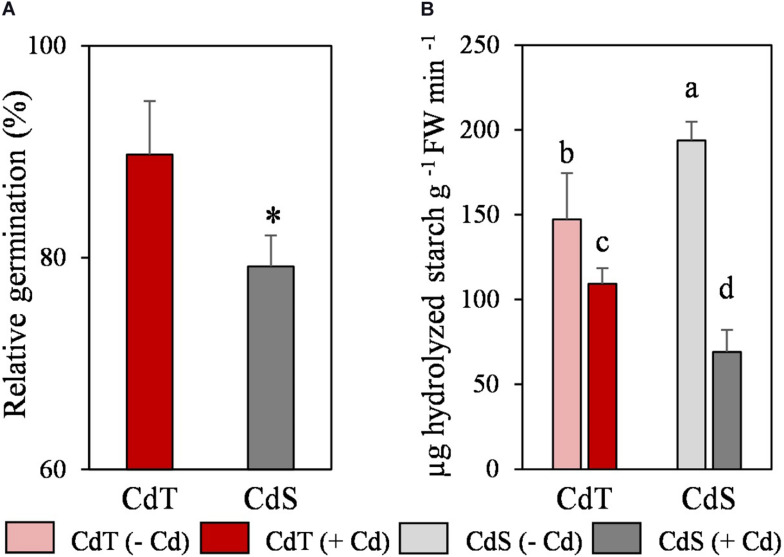
Relative germination in the presence of Cd (200 μM CdCl_2_, 72 h) **(A)** and α-amylase activity in cotyledons **(B)** of *M. truncatula* cultivars CdT (tolerant) and CdS (sensitive). Mean values ± standard deviation are represented. * Indicates significant differences between presence and absence of Cd for each accession [Pearsons’s chi-squared test (χ^2^), *n* = 4]. Different letters indicate significant differences between cultivars and treatments (Tukey HSD test, *p* ≤ 0.05, *n* = 4–6).

We analyzed the effect of Cd on germination and on the α-amylase activity in cotyledons, as an indicator of reserves mobilization. Cd promoted a significant reduction of CdS germination (23%), but it did not affect the germination of CdT (Figure 5A). In the absence of the metal, CdS was able to hydrolyze a significantly higher quantity of starch than CdT (Figure 5B). Cd induced a significant reduction in starch hydrolysis in both accessions, although this reduction was significantly higher in CdS (64%) than in CdT (26%).

To investigate the differences between CdT and CdS in the antioxidant defense response to Cd stress, the expression of some genes that codify for enzymes involved in the antioxidant machinery and PC biosynthesis was studied in seedling roots exposed to 0 or 50 μM CdCl_2_ for 12 h.

**FIGURE 6:**
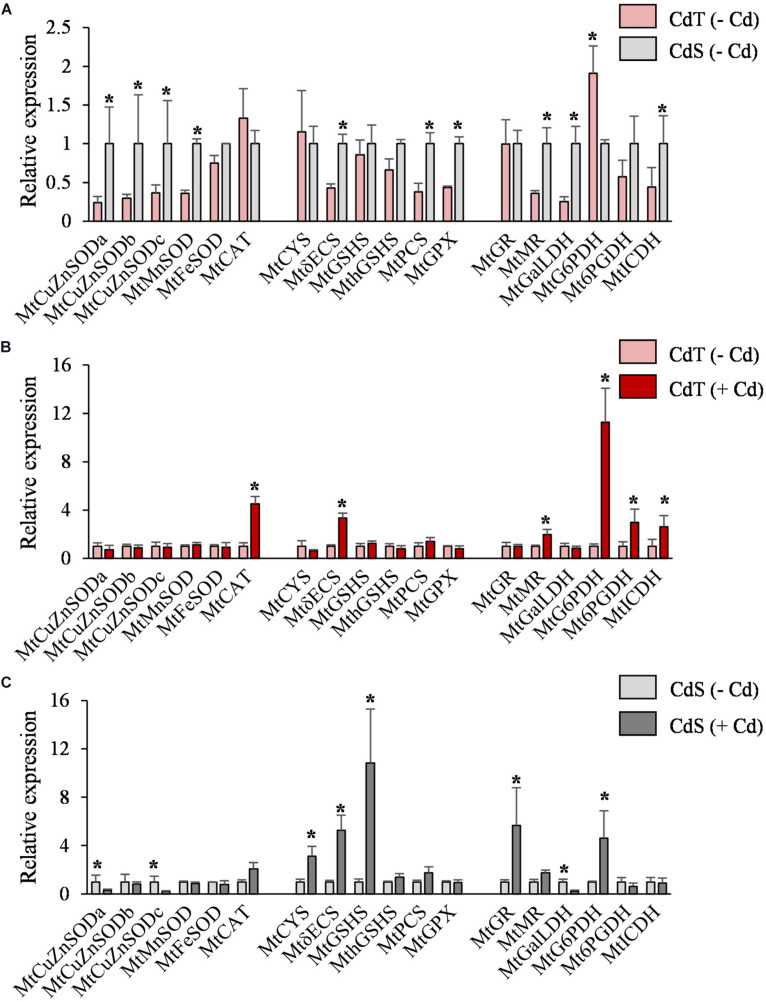
Transcript accumulation of genes involved in the antioxidant machinery and NADPH biosynthesis in seedling roots of *M. truncatula* cultivars CdT (tolerant) and CdS (sensitive) grown in glass containers in the absence or presence of 50 μM CdCl_2_ for 12 h. **(A)** Transcript accumulation for CdT and CdS in absence of Cd. **(B)** Effect of Cd on transcript accumulation in CdT roots. **(C)** Effect of Cd on transcript accumulation in CdS roots. Mean values ± standard deviation are represented (*n* = 4). * Denotes fold-changes greater than two.

In the absence of Cd, the two accessions showed different expression levels for some genes (Figure 6A). Different expression patterns were also observed in CdT and CdS after Cd exposure. The roots of Cd-treated CdT seedlings displayed enhanced expression of *MtCAT*, *Mt*γ*ECS*, and *MtMR* and of the three genes encoding the NADPH-generating enzymes MtG6PDH, Mt6PGDH, and MtICDH, as compared to control unstressed seedling roots (Figure 6B). In contrast, Cd significantly reduced transcript accumulation of *MtCuZnSOD a* and *c* and *MtGalLDH* in CdS roots. Most of the players in GSH and phytochelatin biosynthesis strongly increased their expression in CdS roots (*MtCYS*, *Mt*γ*ECS*, *MtGSHS*, *MtGR*, and *MtG6PDH*) (Figure 6C). The different Cd tolerance of the two accessions might be related to these variations in gene expression.

**FIGURE 7:**
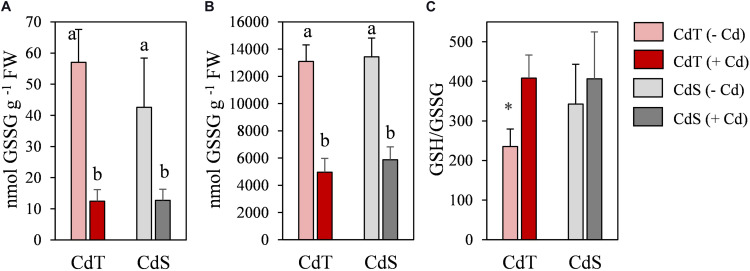
Oxidized glutathione (GSSG) **(A)**, reduced glutathione (GSH) **(B)**, and GSH/GSSG ratio **(C)** in seedling roots of *M. truncatula* cultivars CdT (tolerant) and CdS (sensitive) grown in glass containers in the absence or presence of 50 μM CdCl_2_ for 12 h. Mean values ± standard deviation are represented. Different letters indicate significant differences (Tukey HSD test, *p* ≤ 0.05, *n* = 4). * Denotes significant differences between control and Cd treatment.

To obtain deeper insight into the redox state of the plants, oxidized glutathione (GSSG), reduced glutathione (GSH), and GSH/GSSG ratio were analyzed in CdT and CdS seedling roots (Figure 7). In the absence of Cd, the GSH/GSSG ratio was significantly higher in CdS roots. Cd promoted a significant reduction in glutathione content in both accessions, but the GSH/GSSH ratio increased significantly in Cd-treated CdT roots when compared with control roots, while the ratio did not change upon Cd stress in CdS roots.

**FIGURE 8:**
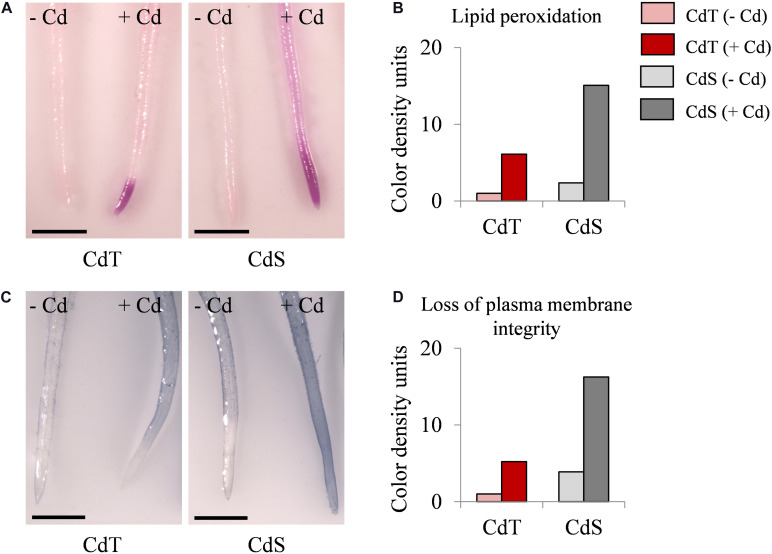
Lipid peroxidation and loss of plasma membrane integrity in seedling roots of *M. truncatula* cultivars CdT (tolerant) and CdS (sensitive) grown in glass containers in the absence or presence of 100 μM CdCl_2_ for 12 h. **(A)** Staining with Schiff’s reagent for lipid peroxidation assessment. **(C)** Staining with Evans Blue for plasma membrane integrity assessment. Quantification of color developed with Schiff’s reagent **(B)** and Evans Blue **(D)** on 1 cm root tips. Scale bar corresponds to 1 mm.

We estimated the oxidative damage caused by Cd on CdT and CdS seedling roots by histochemical detection of lipid peroxidation and plasma membrane integrity. Cd promoted lipid peroxidation in both cultivars but with different intensity and localization (Figures 8A,B). In Cd-treated CdS roots, the staining was found along the entire root and was especially intense in the first 5 mm of the root apex, while in CdT-treated roots, lipid peroxidation was located on the first millimeter of the root apex, suggesting lower oxidative damage than that observed in CdS roots. Cd also had a contrasting effect on the plasma membrane integrity of CdT and CdS seedling roots (Figures 8C,D). Cd-treated CdS roots showed loss of plasma membrane integrity along the whole root. Contrariwise, Cd-treated CdT roots displayed minimal loss of plasma membrane integrity along the root surface and the root apex was not affected (around 4 mm), suggesting again less oxidative damage produced by Cd in the roots of the tolerant cultivar.

**FIGURE 9:**
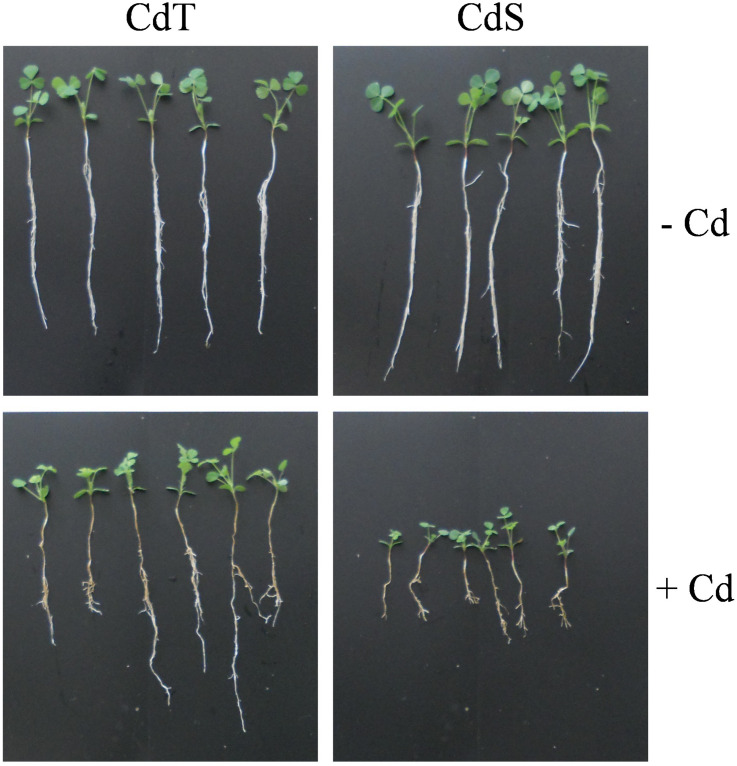
Representative image of the plant growth *M. truncatula* cultivars CdT (tolerant) and CdS (sensitive) grown in pots containing vermiculite with 0 or 100 μM CdCl_2_ for 15 days.

**FIGURE 10:**
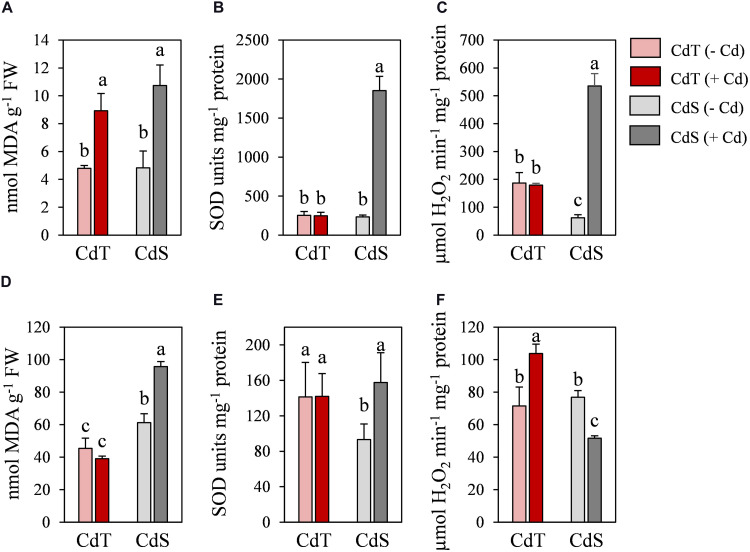
Lipid peroxidation (MDA), and SOD and catalase activities in *M. truncatula* cultivars CdT (tolerant) and CdS (sensitive) grown in pots containing vermiculite with 0 or 100 μM CdCl_2_ for 15 days. **(A–C)** Roots. **(D–F)** Shoots. Different letters indicate significant differences (Tukey HSD test, *p* ≤ 0.05, *n* = 4).

To determine whether CdT and CdS presented differences in their response after a longer exposure to Cd, CdT and CdS plants were grown in the presence or absence of Cd stress for 15 days. Clear differences in plant growth were observed between both cultivars after Cd stress (Figure 9). The lipid peroxidation (MDA content) and the SOD and CAT enzymatic activities were analyzed in roots (Figures 10A–C) and shoots (Figures 10D–F). Cd induced a significant increase of MDA levels in roots of both cultivars. In the absence of Cd, CdS presented a significantly higher MDA content in shoots compared with CdT, and Cd caused a significant increase in MDA content only in CdS shoots.

In the absence of Cd, no significant differences were observed in SOD activity in roots between accessions, while in shoots, CdT presented a significantly higher SOD activity compared with CdS. Cd induced a remarkable increase of the SOD activity in CdS roots and shoots, while CdT SOD activities were unaffected (Figures 10B,E).

In the absence of Cd, CAT activity was significantly higher in CdT roots than in CdS roots (Figures 10C,F). The Cd treatment induced a very significant CAT activity increment in CdS roots and CdT shoots and a significant decrease of CAT activity in CdS shoots (Figure 10F).

## Discussion

Cd toxicity is directly associated to cellular redox imbalance and ROS accumulation. Plant tolerance mechanisms and responses to Cd vary considerably among species and cultivars (Rizwan et al., 2017; Hasanuzzaman et al., 2019; Romero-Puertas et al., 2019). In this work, we evaluated Cd tolerance in a *M. truncatula* germplasm and identified tolerant genotypes. We analyzed several Cd toxicity and Cd tolerance markers in accessions displaying contrasted sensitivity to Cd. Relative root growth (RRG) has been described as a very suitable indicator of heavy metal tolerance (Sledge et al., 2005; Saeidi et al., 2012; García de la Torre et al., 2013; Rahoui et al., 2014). The screening of 258 *M. truncatula* accessions performed here showed a notable intraspecific variability in the response to Cd within *M. truncatula*, which suggests that cultivars with even higher tolerance could be detected by screening larger germplasm collections.

We focused our study on two *M. truncatula* accessions, CdT (tolerant) and CdS (sensitive) that presented similar growth in control conditions, and similar Cd accumulation in their roots and shoots, but remarkable differences in their growth under Cd stress. We analyzed the effect of Cd stress at two stages of plant development: seed germination and vegetative growth (seedlings and 25-day-old plants). Germination is considered a very Cd-sensitive process in comparison with other plant developmental stages (Rahoui et al., 2008), although large differences can exit among plant species and also cultivars (Huybrechts et al., 2019). During germination, reserves mobilization is required to allow plant emergence, and Cd may provoke changes in membrane composition, antioxidant status, and electrical conductivity that might promote nutrient leakages during germination (Sfaxi-Bousbih et al., 2010). Here we showed that both germination and reserves mobilization (starch hydrolysis) were more affected in CdS than in CdT under Cd stress. Significant restrictions in starch mobilization have also been observed in seeds of various Cd-sensitive legume cultivars (Mihoub et al., 2005; Rahoui et al., 2008). Rahoui et al. (2015) suggested that genotype-dependent negative effects of Cd on reserve mobilization, respiration recovery, and nutrient transport during seed germination might explain the different susceptibility to Cd in several lines of *M. truncatula*.

Cadmium uptake causes an imbalance in the nutrient metabolism (uptake, transport, and use) at the root level, probably by competition with the absorption and transport of essential elements, in particular Ca^2+^, Fe^2+^, Mg^2+^, Mn^2+^, Cu^2+^, and Zn^2+^ (DalCorso et al., 2008; Loix et al., 2017; Ismael et al., 2019; Qin et al., 2020). Our results on nutrients contents suggest that Cd could be using different transporters, such as calcium, zinc, and/or iron transporters, as previously described (Thomine et al., 2000; Rodríguez-Serrano et al., 2009; Ismael et al., 2019). Stephens et al. (2011) reported that in *M. truncatula*, Zn uptake was inhibited by Cd. Sulfur is an essential macronutrient, key in protein synthesis, and an important structural component of many co-enzymes and thiol compounds that play important roles in stress tolerance (Ernst et al., 2008). The increase in S content under Cd stress found in CdT roots could contribute to its tolerance, as S assimilation activates the pathway leading to the synthesis of Cys, which is precursor of GSH and other thiol-containing compounds, and it is induced by Cd stress (Baig et al., 2019).

Antioxidant metabolism is critical in Cd tolerance (Terrón-Camero et al., 2020). SODs and CAT represent the first line of response to ROS to maintain the cell redox balance. Enhanced transcript accumulation of SODs after Cd treatment has been described in some *M. truncatula* cultivars, but also the contrary effect in a Cd-susceptible accession (Rahoui et al., 2014). In the present work, we observed repression of *MtCuZnSODa* and *MtCuZnSODc* in CdS seedlings, which could be related to the decrease in zinc content under Cd stress; SOD activity was not affected in CdT, and *CAT* expression was increased in CdT seedlings only. These results evidence differences in the response to Cd of both cultivars.

γ-ECS is a limiting enzyme for GSH synthesis; its activity is GSH regulated and dependent on Cys availability. It has been proposed that a high γ*ECS* expression could compensate GSH deficiency under heavy metal stress (Sobrino-Plata et al., 2014). In CdT seedlings, Cd increased *Mt*γ*ECS* transcript accumulation, but the rest of transcripts related to GSH and PCs biosynthesis (*MtCYS*, *MtGSHS*, and *MtPCS*) remained unaffected. On the contrary, in CdS, Cd provoked an increase in transcript accumulation of *MtCYS*, *Mt*γ*ECS*, *MtGSHS*, as well as *MtGR*, which codes for a glutathione reductase, a significant enzyme in maintaining redox homeostasis via a correct GSH/GSSG ratio. This suggest that Cd tolerance is not so much dependent on phytochelatin quelation in tolerant plants, which is a common response to Cd stress in sensitive species as previously reported (Schat et al., 2002).

Glutathione plays a central role as chelator, antioxidant, and signaling molecule (Jozefczak et al., 2012). Cd caused a reduction in GSSG and GSH contents in the roots of both accessions and an increase in GSH/GSSG ratio in CdT, but not in CdS. These results are in agreement with the decrease in glutathione content without changes in GSH/GSSG ratio reported for a Cd-sensitive *M. sativa* cultivar (Sobrino-Plata et al., 2009).

NADPH is important in the defense against oxidative stress (Foyer and Noctor, 2011). It has been reported that Cd tolerance might be more dependent on NADPH availability than on its antioxidant capacity (León et al., 2002). The upregulation of gene expression and increased activities of NADPH-recycling dehydrogenases have been reported in *M. truncatula* nodules and soybean roots in response to Cd (Marino et al., 2013; Pérez-Chaca et al., 2014). The enhanced expression of the genes encoding three NADPH-recycling enzymes in CdT strongly suggests that it might represent an important defense mechanism, which appears to be absent in CdS.

Following Cd exposure, oxidative stress is considered as the origin of damage in plant tissues (Schützendübel et al., 2002; Rahoui et al., 2017). Cd-induced lipid peroxidation has been reported in *M. truncatula* (Sobrino-Plata et al., 2009; Rahoui et al., 2017). In CdS seedling roots, Cd produced higher lipid peroxidation and loss of cell membrane integrity than in CdT. Taken together, our results on seedlings indicate that Cd promoted a more severe oxidative damage in CdS than in CdT, which was able to better neutralize ROS production. In agreement with our results, a correlation between Cd tolerance and low oxidative stress at the seedling stage has been described in tolerant *M. truncatula* accessions (Rahoui et al., 2014).

Metal tolerance and/or toxicity also depend on the plant developmental stage. We analyzed the differences in tolerance of 25-day-old CdT and CdS plants after a long exposure to Cd. Lipid peroxidation (MDA content) and SOD and CAT activities were determined in roots and shoots. MDA content was unaffected in CdT shoots, and SOD activity was also not affected in CdT roots or shoots. Catalase activity significantly increased in CdT shoots, which could explain why lipid peroxidation did not increase in CdT shoots. In CdS roots, catalase activity increased, while a decrease was observed in shoots. The consequent accumulation of H_2_O_2_ seems to be key in inducing growth reduction associated to Cd stress (Schützendübel et al., 2002).

**FIGURE 11:**
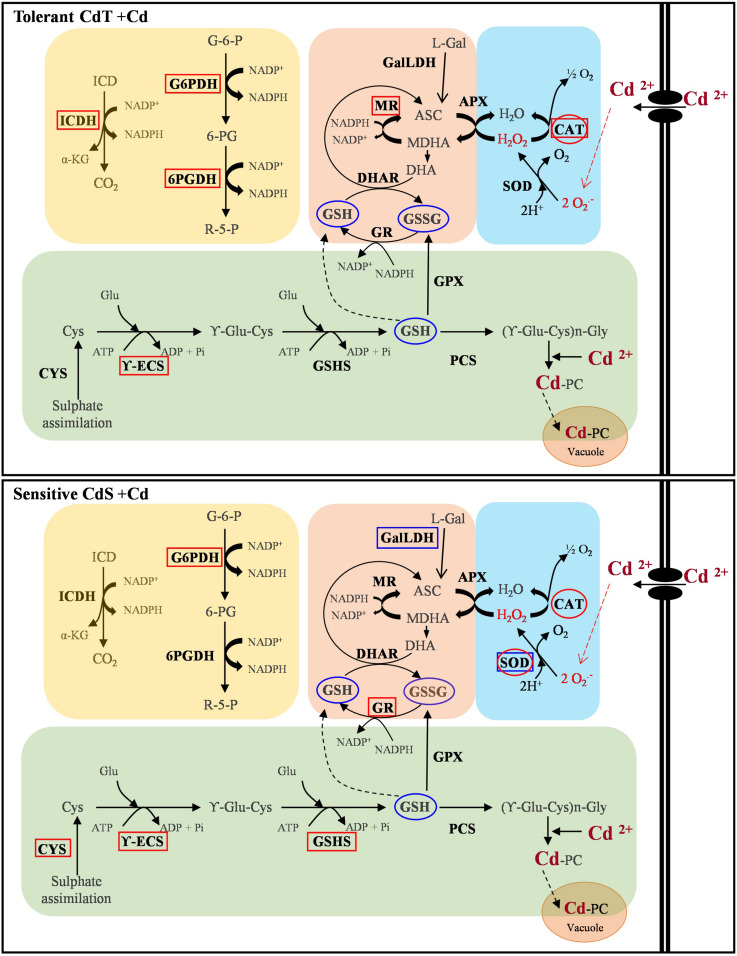
Schematic overview of the contrasting responses observed in *M. truncatula* cultivars CdT (tolerant) and CdS (sensitive) upon Cd stress relative to gene expression, enzyme activity, and antioxidant content. Analyzed parameter is in bold letter. Rectangles denote a Cd effect on the expression of the gene encoding the enzyme. Ovals indicate a Cd effect on the enzymatic activity or content of non-enzymatic antioxidants. Red and blue colors denote increase and decrease, respectively, as compared with non-Cd treated plants. ASC, ascorbate; CAT, catalase; Cys, cysteine; CYS, cysteine synthase; DHA, dehydroascorbate; DHAR, dehydroascorbate reductase; GalLDH, galactono-1,4-lactone dehydrogenase; Glu, glutamic; G6P, glucose-6-phosphate; G6PDH, glucose-6-phosphate dehydrogenase; GPX, glutathione peroxidase; GR, glutathione reductase; GSSG, oxidized glutathione; GSH, reduced glutathione; GSHS, glutathione synthetase; ICD, isocitrate; ICDH, isocitrate dehydrogenase; L-Gal, L-galactono-lactone; MDHA, monodehydroascorbate; MR, monodehydroascorbate reductase; PCS, phytochelatin synthase; R5P, ribulose-5-phosphate; SOD, superoxide dismutase; 6PGDH, 6-phosphogluconate dehydrogenase; γ-ECS, γ-glutamyl-cysteine synthetase; α-KG, α-ketoglutarate.

Figure 11 summarizes our results on gene expression, enzyme activity, and antioxidant content. A contrasting behavior of the two cultivars can be observed.

In summary, CdT was more tolerant to Cd stress than CdS at all developmental stages studied. Both cultivars shared an increased CAT activity. In CdS, SOD increased in response to primary oxidative stress, while it did not seem to play a crucial role in CdT response. Their additional response mechanisms were different. CdT ensured availability of NADPH by increasing expression of NADPH-recycling enzymes, and, unexpectedly, presented no induction of the PC biosynthesis pathway. On the contrary, CdS upregulated the expression of three of the four genes involved in PC biosynthesis, but not those responsible for NADPH recycling. NADPH availability appears as the crucial factor in Cd tolerance in *M. truncatula*. PCs represent a common response to heavy metals, but depending on the stress level, they might not be sufficient. Conversely, when alternative factors are activated, PCs increase might not be necessary.

Our results point at the possibility of identifying highly tolerant cultivars with additional traits, such as high or low accumulation and translocation. The number of *M. truncatula* accessions available in different collections worldwide is above 6,000. Forage legumes present an important potential to be used in heavy-metal phytoremediation. Cultivars with high levels of the enzymes involved in NADPH production could be used to transfer the tolerance trait to cultivated *M. truncatula*, alfalfa, or other legumes. Additionally, a collection of *M. truncatula* mutant lines is available from the Noble Research Institute^3^, and there are over 300 fully sequenced *M. truncatula* cultivars that can be obtained from the Medicago Hapmap Project Germplasm^4^. These resources will facilitate the identification and analysis of genes and loci involved in Cd tolerance and accumulation, and a better understanding of Cd tolerance and detoxifying mechanisms.

## Data Availability Statement

The original contributions presented in the study are included in the article and the Supplementary Material. Further inquiries can be directed to the authors.

## Author Contributions

VSGT, TCP, JJP, and MML conceived the experiments. VSGT performed the experiments and wrote the first draft. TCP contributed to the experimental part. JJP and MML supervised and contributed to the experiments. TCP, JJP, and MML wrote the last version of the manuscript. All authors read and approved the submitted version.

## Conflict of Interest

The authors declare that the research was conducted in the absence of any commercial or financial relationships that could be construed as a potential conflict of interest.

## Footnotes

1 https://bioinfo.ut.ee/primer3-0.4.0/

2 http://imagej.nih.gov/ij/

3 https://www.noble.org/

4 http://www.medicagohapmap.org/
