# Supplementary material for: Cadmium-Tolerant and -Sensitive Cultivars Identified by Screening of Medicago truncatula Germplasm Display Contrasting Responses to Cadmium Stress
Source: Front Plant Sci. 2021 Mar 11;12:595001. doi: 10.3389/fpls.2021.595001 (PMC7991585; doi:10.3389/fpls.2021.595001)

Supplementary Figure S1. Relative root growth of *M. truncatula* cv. Parabinga seedlings exposed to different Cd concentrations for varying times. Mean values are shown (n = 10–30) and the bars indicate the standard errors.

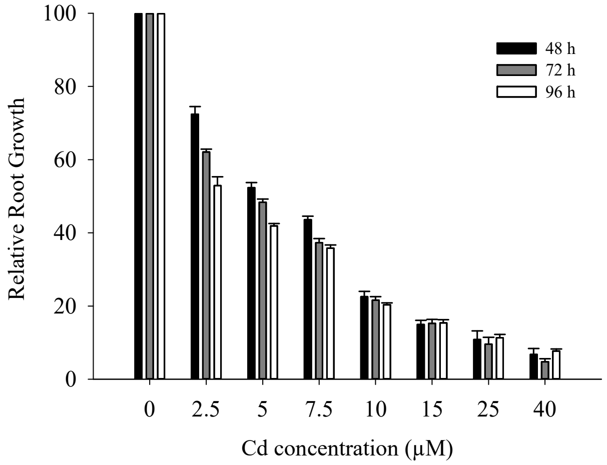

Supplement: Supplementary file 1 [file Data_Sheet_1.pdf]
