# Supplementary material for: Cadmium-Tolerant and -Sensitive Cultivars Identified by Screening of Medicago truncatula Germplasm Display Contrasting Responses to Cadmium Stress
Source: Front Plant Sci. 2021 Mar 11;12:595001. doi: 10.3389/fpls.2021.595001 (PMC7991585; doi:10.3389/fpls.2021.595001)
Supplement: Supplementary file 2 [file Table_1.pdf]

Supplementary Table 1. Primer sequences used for gene expression analysis and accession number (Acc. N.) or locus names of genes in this study. Primer3 software (https://bioinfo.ut.ee/primer3-0.4.0/) was used for the primer design; Acc. N. and locus names were obtained from available genomic sequences at the National Center for Biotechnology Information (http://www.ncbi.nlm.nih.gov) and the *Medicago Truncatula* Genome Project v4.0 (http://www.jcvi.org/medicago/).

Gene	Forward 5'-3'	Reverse 5'-3'	Acc. N. or Locus Name
MtACTIN11	GGTCCATCAATTGTCCACAG	ATGCTTCTGGCTGATTCACA	XM_003621972
MtCuZnSODa	TGGTTGGCTTGACTCCAGTA	AATGGCAGTAGCCCATCAAG	Medtr4g057240
MtCuZnSODb	CTCACCGGACCAAACTCAAT	AGCTACTCTGCCACCAGCAT	Medtr7g114240
MtCuZnSODc	GAAGGGCTGTCGTTGTTCAT	TCCAATGATACCGCATGCTA	Medtr6g029200
MtFeSOD	TCAATTCCAAATTACTGACTTTGTG	ATATTCAGGACGCCGATTCT	Medtr1g048990
MtMnSOD	TAAAGTGCTGCTTGGTGTGG	CTGCGCCCTAAGGAAAGTCT	Medtr3g094250
CAT	GCACCCGACAGGCAAGATAGATT	CGAGTCACGTACATGGAGTTTA	XM_013606823
MtCYS	TCCTCTGTGCTATTTGAGTCAGTGA	AATGATTCAGGGCTCGAAAGTC	XM_003610676
MtγECS	GCGTCTTGCTTTTCTTTCACATT	TTCTACCCAAAAAAGGTGGCTTAT	AF041340
MtGSHS	CATTACCATTTGAGCCTCCTGTT	TGTGGCCTCCCTTTCAATTT	XM_003626276
MthGSHS	CACATGATGAAACCAACTCATTGA	AACATGTCATGGGAGAAAGTAAAAGA	AF194421
MtPCS	ATCTCGGTGCTCCTTCCTCT	CAGAAGCAGAATTTTGGTGAC	Medtr7g097190
MtGPX	CCAACAACCTCTCCTTTTCAAATT	CTCTGATTTTGCTCATTCCTGAATAT	XM_003588826
MtGR	CCCAAGACGAATTTGTAAACACAT	CCCAATAAAAGAAACATACAAACAAGTC	BT149819
MtMR	CTACAGCTGGGGTCGTTCTT	AAGCCCTTAACCACAAATCG	Medtr7g034715
MtGalLDH	CTCCAAGCAAGGCTGAGAAA	GCTTTTCCAACTTGTTGTTCG	Medtr1g050360
MtG6PDH	ACAAGCCGGGAAGTAGAGGT	TGGTCACTCTATAAGGTAGGAGGAA	Medtr7g037440
Mt6PGDH	CGAGACTACTTCGGTGCTCA	TCTAAATCCTCGACTGCTTGG	Medtr7g017900
MtICDH	TGGCTGCTGATCTTAAAACAAA	TGCTTCGTTCCCCTTTCTTA	Medtr5g077070

MtACTIN11: actin; *MtCuZnSODa*, *MtCuZnSODb*, *MtCuZnSODc*: cooper/zinc superoxide dismutases; *MtFeSOD*: ferric SOD; *MtMnSOD*: manganese SOD; *CAT*: catalase; *MtCYS*: cysteine synthase; *MtγECS*: γ-glutamyl-cysteine synthetase; *MtGSHS*: glutathione synthetase; *MthGSHS*: homoglutathione synthetase; *MtPCS*: phytochelatin synthase; *MtGPX*: glutathione peroxidase; *MtGR*: glutathione reductase; *MtMR*: monodehydroascorbate reductase; *MtGalLDH*: L-galactono-1,4-lactone dehydrogenase; *MtG6PDH*: glucose-6-phosphate dehydrogenase; *Mt6PGDH*: 6-phosphogluconate dehydrogenase; *MtICDH*: isocitrate dehydrogenase.
