# Supplementary material for: Cadmium-Tolerant and -Sensitive Cultivars Identified by Screening of Medicago truncatula Germplasm Display Contrasting Responses to Cadmium Stress
Source: Front Plant Sci. 2021 Mar 11;12:595001. doi: 10.3389/fpls.2021.595001 (PMC7991585; doi:10.3389/fpls.2021.595001)
Supplement: Supplementary file 3 [file Table_2.pdf]

Supplementary Table 2. Relative root growth (RRG) and increase of root length of 258 *M. truncatula* accessions after 48 h in a miniaturized hydroponic system in the absence or presence of 10μM $CdCl_2$. Asterisks indicate significant differences in root length increment between control and Cd treatments for each accession (ANOVA, $p \leq 0.05$, $n \geq 10$). Mean ± Standard deviation are indicated for root length increment.

Cultivar	RRG	Δ Root Length (cm)		Cultivar	RRG	Δ Root Length (cm)	
		Cd	Control			Cd	Control
**PI 190082**	29.87*	0.46 ± 0.11	1.54 ± 0.16	**PI 577639**	23.68*	0.55 ± 0.14	2.32 ± 0.35
**PI 190083**	37.99*	0.65 ± 0.20	1.58 ± 0.17	**PI 577640**	29.20*	0.50 ± 0.13	1.71 ± 0.22
**PI 190084**	35.77*	0.88 ± 0.18	2.46 ± 0.82	**PI 577641**	22.22*	0.40 ± 0.19	1.80 ± 0.12
**PI 190086**	45.90*	0.62 ± 0.10	1.35 ± 0.11	**PI 577642**	42.19*	1.33 ± 0.17	3.16 ± 0.44
**PI 190089**	27.18*	0.56 ± 0.10	2.06 ± 0.16	**PI 577643**	49.37*	1.02 ± 0.19	2.06 ± 0.23
**PI 190090**	33.44*	0.94 ± 0.11	2.81 ± 0.07	**PI 577646**	39.73*	0.97 ± 0.06	2.43 ± 0.11
**PI 197341**	26.42*	0.42 ± 0.23	1.59 ± 0.15	**PI 641406**	50.38*	0.74 ± 0.09	1.47 ± 0.17
**PI 197358**	59.61*	1.21 ± 0.09	2.03 ± 0.20	**PI 641407**	20.50*	0.51 ± 0.11	2.50 ± 0.20
**PI 197360**	38.89*	0.77 ± 0.16	1.98 ± 0.21	**PI 641408**	50.67*	0.38 ± 0.06	0.75 ± 0.08
**PI 197361**	46.26*	0.77 ± 0.25	1.65 ± 0.25	**PI 641409**	25.73*	0.69 ± 0.16	2.67 ± 0.16
**PI 199257**	8.93*	0.14 ± 0.08	1.60 ± 0.20	**PI 641410**	67.67*	1.07 ± 0.42	1.58 ± 0.18
**PI 239876**	23.14*	0.56 ± 0.25	2.42 ± 0.09	**PI 641411**	19.15*	0.45 ± 0.12	2.35 ± 0.17
**PI 239877**	25.00*	0.70 ± 0.08	2.80 ± 0.21	**PI 641412**	29.57*	0.93 ± 0.24	3.14 ± 0.50
**PI 239878**	36.70*	0.47 ± 0.08	1.27 ± 0.20	**PI 641413**	22.48*	0.54 ± 0.09	2.42 ± 0.39
**PI 243884**	39.22*	0.67 ± 0.08	1.70 ± 0.15	**PI 660385**	31.82*	0.23 ± 0.06	0.73 ± 0.05
**PI 244285**	19.64*	0.37 ± 0.06	1.87 ± 0.11	**PI 660498**	43.87*	1.03 ± 0.15	2.35 ± 0.08
**PI 283661**	28.47*	0.47 ± 0.16	1.60 ± 0.22	**PI 660361**	51.90*	0.68 ± 0.17	1.31 ± 0.09
**PI 283662**	47.73*	0.70 ± 0.16	1.46 ± 0.18	**PI 660364**	33.33*	0.57 ± 0.06	1.70 ± 0.10
**PI 284123**	37.29*	0.63 ± 0.14	1.69 ± 0.15	**PI 660365**	74.29*	1.04 ± 0.09	1.40 ± 0.12
**PI 292434**	37.88*	0.58 ± 0.10	1.54 ± 0.13	**PI 660367**	25.71*	0.75 ± 0.05	2.92 ± 0.17

**PI 292436**	28.69*	0.66 ± 0.14	2.31 ± 0.27	**PI 660370**	36.43*	0.78 ± 0.15	2.15 ± 0.20
**PI 295607**	48.74*	1.03 ± 0.09	2.12 ± 0.18	**PI 660371**	27.27*	0.66 ± 0.11	2.42 ± 0.08
**PI 319045**	58.46*	1.27 ± 0.06	2.17 ± 0.20	**PI 660372**	48.65*	1.08 ± 0.15	2.22 ± 0.22
**PI 319051**	24.51*	0.58 ± 0.12	2.36 ± 0.17	**PI 660373**	30.59*	0.52 ± 0.13	1.70 ± 0.20
**PI 384633**	17.91*	0.40 ± 0.17	2.23 ± 0.08	**PI 660375**	35.09*	0.67 ± 0.12	1.90 ± 0.12
**PI 384634**	9.75*	0.14 ± 0.15	1.41 ± 0.16	**PI 660376**	14.53*	0.34 ± 0.15	2.34 ± 0.26
**PI 384635**	18.68*	0.24 ± 0.19	1.27 ± 0.19	**PI 660378**	25.14*	0.73 ± 0.14	2.92 ± 0.16
**PI 384636**	54.64*	0.53 ± 0.11	0.97 ± 0.15	**PI 660379**	43.21*	1.00 ± 0.08	2.31 ± 0.19
**PI 384645**	25.93*	0.23 ± 0.05	0.90 ± 0.15	**PI 660380**	22.10*	0.57 ± 0.18	2.58 ± 0.70
**PI 384647**	36.34*	0.86 ± 0.24	2.36 ± 0.15	**PI 660383**	40.00*	1.00 ± 0.08	2.50 ± 0.15
**PI 384648**	23.78*	0.49 ± 0.08	2.05 ± 0.18	**PI 660384**	23.17*	0.63 ± 0.06	2.73 ± 0.05
**PI 384649**	36.84*	0.88 ± 0.17	2.37 ± 0.09	**PI 660386**	40.63*	0.52 ± 0.08	1.28 ± 0.13
**PI 384650**	44.87*	0.70 ± 0.16	1.56 ± 0.11	**PI 660387**	32.24*	0.54 ± 0.13	1.80 ± 0.20
**PI 384655**	36.30*	0.59 ± 0.22	1.62 ± 0.13	**PI 660388**	24.84*	0.63 ± 0.05	2.55 ± 0.16
**PI 384656**	28.65*	0.47 ± 0.21	1.65 ± 0.24	**PI 660389**	13.24*	0.30 ± 0.08	2.26 ± 0.60
**PI 384660**	24.31*	0.39 ± 0.11	1.60 ± 0.11	**PI 660390**	33.82*	0.66 ± 0.13	1.94 ± 0.09
**PI 384662**	53.25*	0.68 ± 0.19	1.28 ± 0.21	**PI 660391**	48.03*	0.73 ± 0.13	1.52 ± 0.19
**PI 384664**	20.65*	0.48 ± 0.16	2.30 ± 0.15	**PI 660392**	13.04*	0.20 ± 0.15	1.53 ± 0.21
**PI 384665**	29.46*	0.41 ± 0.08	1.40 ± 0.12	**PI 660394**	16.49*	0.40 ± 0.17	2.42 ± 0.22
**PI 464815**	30.08*	1.00 ± 0.08	3.32 ± 0.09	**PI 660395**	23.44*	0.38 ± 0.21	1.60 ± 0.17
**PI 464816**	20.96*	0.50 ± 0.11	2.38 ± 0.21	**PI 660396**	58.62*	0.57 ± 0.05	0.97 ± 0.05
**PI 469099**	51.72*	0.75 ± 031	1.45 ± 0.34	**PI 660397**	40.00*	1.00 ± 0.09	2.50 ± 0.14
**PI 469100**	27.82*	0.51 ± 0.08	1.84 ± 0.15	**PI 660398**	36.29*	0.83 ± 0.11	2.28 ± 0.22
**PI 469102**	42.74*	0.67 ± 0.08	1.56 ± 0.11	**PI 660399**	23.49*	0.50 ± 0.19	2.13 ± 0.17

**PI 493295**	25.75*	0.56 ± 0.24	2.17 ± 0.11	**PI 660400**	14.88*	0.30 ± 0.12	2.02 ± 0.17
**PI 493296**	20.62*	0.25 ± 0.12	1.21 ± 0.14	**PI 660401**	49.53*	0.76 ± 0.11	1.52 ± 0.16
**PI 493297**	40.10*	0.55 ± 0.08	1.37 ± 0.21	**PI 660402**	25.91*	0.56 ± 0.34	2.15 ± 0.10
**PI 505438**	31.98*	0.62 ± 0.12	2.05 ± 0.32	**PI 660403**	4.24*	0.14 ± 0.05	3.30 ± 0.12
**PI 516923**	19.56*	0.38 ± 0.19	1.96 ± 0.08	**PI 660404**	28.17*	0.67 ± 0.13	2.36 ± 0.27
**PI 516924**	40.58*	0.56 ± 0.13	1.38 ± 0.17	**PI 660405**	37.78*	0.43 ± 0.14	1.12 ± 0.11
**PI 516925**	37.14*	0.65 ± 0.06	1.75 ± 0.19	**PI 660406**	20.03*	0.52 ± 0.13	2.58 ± 0.27
**PI 516926**	27.34*	0.56 ± 0.16	2.04 ± 0.21	**PI 660407**	83.33	1.00 ± 0.18	1.20 ± 0.11
**PI 516927**	16.11*	0.27 ± 0.07	1.65 ± 0.20	**PI 660408**	42.86*	0.70 ± 0.12	1.63 ± 0.25
**PI 516928**	20.16*	0.42 ± 0.21	2.07 ± 0.20	**PI 660409**	28.07*	0.64 ± 0.05	2.28 ± 0.14
**PI 516929**	85.87	1.32 ± 0.20	1.53 ± 0.13	**PI 660410**	18.21*	0.42 ± 0.12	2.92 ± 0.20
**PI 516930**	30.33*	0.43 ± 0.10	1.42 ± 0.11	**PI 660411**	66.18	1.13 ± 0.10	1.70 ± 0.50
**PI 516931**	29.03*	0.68 ± 0.10	2.32 ± 0.09	**PI 660412**	48.91*	0.75 ± 0.10	1.53 ± 0.16
**PI 516933**	90.00	1.13 ± 0.15	1.25 ± 0.12	**PI 660414**	39.61*	1.22 ± 0.08	3.08 ± 0.16
**PI 516934**	44.44*	0.73 ± 0.21	1.65 ± 0.10	**PI 660415**	49.40*	1.19 ± 0.23	2.40 ± 0.15
**PI 516935**	18.35*	0.43 ± 0.15	2.57 ± 0.12	**PI 660417**	32.18*	0.47 ± 0.25	1.45 ± 0.27
**PI 516936**	50.53*	0.96 ± 0.12	1.90 ± 0.19	**PI 660418**	26.99*	0.56 ± 0.18	2.07 ± 0.09
**PI 516937**	45.45*	0.84 ± 0.14	1.84 ± 0.33	**PI 660419**	43.64*	0.48 ± 0.11	1.43 ± 0.05
**PI 516938**	47.30*	0.88 ± 0.17	1.85 ± 0.17	**PI 660420**	41.76*	0.76 ± 0.05	1.82 ± 0.08
**PI 516939**	55.93*	0.83 ± 0.05	1.47 ± 0.20	**PI 660421**	11.20*	0.28 ± 0.13	2.50 ± 0.24
**PI 516940**	35.34*	0.41 ± 0.13	1.16 ± 0.25	**PI 660422**	28.49*	0.52 ± 0.13	1.70 ± 0.38
**PI 516942**	28.47*	0.62 ± 0.10	2.18 ± 0.12	**PI 660423**	41.79*	0.70 ± 0.08	1.67 ± 0.05
**PI 516943**	32.43*	0.84 ± 0.10	2.59 ± 0.19	**PI 660424**	18.71*	0.31 ± 0.08	1.67 ± 0.25
**PI 516944**	18.24*	0.44 ± 0.23	2.41 ± 0.19	**PI 660425**	26.73*	0.90 ± 0.02	3.36 ± 0.22

**PI 516947**	33.61*	0.79 ± 0.07	2.33 ± 0.25	**PI 660426**	53.49*	0.46 ± 0.15	0.86 ± 0.37
**PI 516948**	48.75*	0.65 ± 0.09	1.33 ± 0.12	**PI 660428**	23.28*	0.54 ± 0.05	2.32 ± 0.14
**PI 516949**	27.72*	0.70 ± 0.10	2.52 ± 0.09	**PI 577646**	36.05*	0.88 ± 0.15	2.45 ± 0.25
**PI 516950**	44.25*	0.59 ± 0.09	1.33 ± 0.22	**PI 660429**	20.00*	0.32 ± 0.04	1.60 ± 0.24
**PI 517256**	35.09*	0.67 ± 0.06	1.90 ± 0.36	**PI 660430**	29.60*	0.46 ± 0.11	1.56 ± 0.22
**PI 517257**	25.25*	0.43 ± 0.17	1.68 ± 0.19	**PI 660432**	34.12*	0.58 ± 0.08	1.70 ± 0.10
**PI 535543**	29.32*	0.98 ± 0.10	3.32 ± 0.10	**PI 660433**	32.79*	1.00 ± 0.14	3.05 ± 0.17
**PI 535546**	72.92*	0.50 ± 0.10	0.68 ± 0.06	**PI 660434**	35.35*	0.58 ± 0.08	1.65 ± 0.10
**PI 535547**	25.49*	0.52 ± 0.08	2.04 ± 0.15	**PI 660435**	33.64*	1.04 ± 0.30	3.10 ± 0.25
**PI 535548**	24.49*	0.60 ± 0.29	2.45 ± 0.20	**PI 660436**	28.99*	0.61 ± 0.16	2.11 ± 0.21
**PI 535549**	44.63*	0.68 ± 0.22	1.51 ± 0.20	**PI 660437**	39.47*	1.00 ± 0.11	2.53 ± 0.24
**PI 535550**	29.05*	0.68 ± 0.10	2.33 ± 0.18	**PI 660438**	29.01*	0.44 ± 0.28	1.52 ± 0.29
**PI 535552**	29.27*	0.40 ± 0.09	1.36 ± 0.21	**PI 660439**	45.52*	0.68 ± 0.13	1.49 ± 0.22
**PI 535554**	33.33*	0.80 ± 0.11	2.40 ± 0.30	**PI 660440**	38.14*	1.19 ± 0.14	3.12 ± 0.32
**PI 535614**	66.72*	0.61 ± 0.14	0.91 ± 0.08	**PI 660441**	31.03*	0.60 ± 0.12	1.93 ± 0.35
**PI 535615**	82.59*	0.78 ± 0.08	0.94 ± 0.10	**PI 660442**	47.59*	0.86 ± 0.13	1.81 ± 0.22
**PI 535616**	39.17*	0.28 ± 0.16	0.73 ± 0.09	**PI 660443**	39.18*	0.95 ± 0.79	2.42 ± 0.29
**PI 535618**	80.33*	0.54 ± 0.05	0.68 ± 0.08	**PI 660444**	42.06*	0.75 ± 0.10	1.78 ± 0.16
**PI 535619**	122.86*	1.43 ± 0.15	1.16 ± 0.05	**PI 660445**	40.60*	0.77 ± 0.20	1.90 ± 0.20
**PI 535622**	84.00	0.53 ± 0.09	0.62 ± 0.11	**PI 660446**	31.65*	0.50 ± 0.11	1.58 ± 0.10
**PI 535648**	16.13*	0.33 ± 0.13	2.06 ± 0.05	**PI 660449**	16.25*	0.52 ± 0.04	3.2 ± 0.83
**PI 535650**	88.65*	2.05 ± 0.14	2.31 ± 0.09	**PI 660450**	18.66*	0.50 ± 0.20	2.68 ± 0.13
**PI 535652**	38.96*	0.64 ± 0.16	1.65 ± 0.31	**PI 660451**	58.49*	0.78 ± 0.13	1.32 ± 0.09
**PI 535739**	44.44*	0.67 ± 0.16	1.50 ± 0.08	**PI 660452**	34.38*	0.66 ± 0.11	1.92 ± 0.08

**PI 535752**	30.89*	0.48 ± 0.10	1.54 ± 0.11	**PI 660453**	66.94*	1.01 ± 0.08	1.51 ± 0.27
**PI 564941**	22.64*	0.40 ± 0.08	1.76 ± 0.15	**PI 660454**	37.93*	0.44 ± 0.05	1.16 ± 0.15
**PI 566886**	34.48*	0.50 ± 0.09	1.65 ± 0.33	**PI 660455**	43.33*	1.04 ± 0.13	2.40 ± 0.21
**PI 566887**	37.35*	1.03 ± 0.12	2.76 ± 0.23	**PI 660456**	44.25*	0.71 ± 0.17	1.61 ± 0.21
**PI 566890**	46.43*	0.98 ± 0.10	2.10 ± 0.08	**PI 660457**	31.43*	0.47 ± 0.13	1.50 ± 0.19
**PI 577434**	39.75*	0.64 ± 0.08	1.61 ± 0.05	**PI 660458**	41.89*	0.52 ± 0.16	1.23 ± 0.22
**PI 577597**	53.33*	0.80 ± 0.17	1.50 ± 0.20	**PI 660459**	23.49*	0.58 ± 0.12	2.48 ± 0.14
**PI 577600**	66.67*	0.85 ± 0.05	1.27 ± 0.08	**PI 660461**	36.36*	0.60 ± 0.20	1.65 ± 0.23
**PI 577601**	22.69*	0.49 ± 0.09	2.16 ± 0.16	**PI 660462**	44.67*	0.68 ± 0.10	1.52 ± 0.29
**PI 577603**	35.82*	0.89 ± 0.11	2.47 ± 0.23	**PI 660463**	16.94*	0.30 ± 0.20	1.77 ± 0.12
**PI 577604**	35.88*	0.87 ± 0.08	2.43 ± 0.37	**PI 660464**	44.71*	0.76 ± 0.13	1.70 ± 0.15
**PI 577607**	35.45*	0.79 ± 0.16	2.22 ± 0.73	**PI 660465**	43.29*	1.01 ± 0.15	2.34 ± 0.25
**PI 577608**	23.20*	0.58 ± 0.08	2.50 ± 0.21	**PI 660466**	48.35*	0.73 ± 0.14	1.52 ± 0.21
**PI 577609**	24.06*	0.53 ± 0.10	2.21 ± 0.11	**PI 660467**	42.95*	0.91 ± 0.11	2.13 ± 0.11
**PI 577610**	33.83*	0.90 ± 0.10	2.66 ± 0.23	**PI 660469**	31.06*	1.03 ± 0.10	3.30 ± 0.16
**PI 577611**	24.18*	0.63 ± 0.10	2.60 ± 0.26	**PI 660472**	16.43*	0.38 ± 0.08	2.33 ± 0.17
**PI 577612**	40.41*	0.47 ± 0.11	1.16 ± 0.13	**PI 660474**	52.94*	0.90 ± 0.08	1.70 ± 11
**PI 577613**	39.53*	0.85 ± 0.13	2.15 ± 0.12	**PI 660475**	27.03*	0.50 ± 0.08	1.85 ± 0.10
**PI 577614**	21.65*	0.30 ± 0.12	1.38 ± 0.10	**PI 660476**	44.62*	0.73 ± 0.09	1.62 ± 0.04
**PI 577615**	20.41*	0.50 ± 0.14	2.45 ± 0.19	**PI 660477**	23.49*	0.78 ± 0.08	3.32 ± 0.16
**PI 577616**	17.88*	0.39 ± 0.07	2.16 ± 0.13	**PI 660478**	28.83*	0.53 ± 0.15	1.85 ± 0.66
**PI 577617**	28.26*	0.65 ± 0.19	2.30 ± 0.25	**PI 660479**	5.51*	0.20 ± 0.23	3.63 ± 0.34
**PI 577618**	18.61*	0.38 ± 0.16	2.03 ± 0.42	**PI 660480**	22.52*	0.50 ± 0.32	2.22 ± 0.57
**PI 577619**	33.06*	0.67 ± 0.18	2.02 ± 0.26	**PI 660481**	23.42*	0.62 ± 0.17	2.63 ± 0.08

**PI 577620**	28.70*	0.69 ± 0.09	2.40 ± 0.31	**PI 660482**	28.13*	0.45 ± 0.29	1.60 ± 0.23
**PI 577623**	31.75*	0.40 ± 0.17	1.26 ± 0.18	**PI 660483**	15.94*	0.37 ± 0.12	2.30 ± 0.17
**PI 577624**	11.33*	0.34 ± 0.17	3.00 ± 0.1	**PI 660484**	35.90*	0.80 ± 0.10	2.23 ± 0.14
**PI 577625**	12.50*	0.30 ± 0.14	2.40 ± 0.10	**PI 660486**	19.39*	0.32 ± 0.08	1.65 ± 0.18
**PI 577626**	21.08*	0.54 ± 0.12	2.55 ± 0.13	**PI 660487**	43.92*	1.19 ± 0.11	2.70 ± 0.17
**PI 577627**	35.69*	0.58 ± 0.04	1.62 ± 0.15	**PI 660488**	25.49*	0.58 ± 0.20	2.26 ± 0.26
**PI 577628**	10.77*	0.14 ± 0.15	1.30 ± 0.24	**PI 660490**	38.97*	0.88 ± 0.16	2.26 ± 0.10
**PI 577629**	17.02*	0.38 ± 0.13	2.22 ± 0.16	**PI 660491**	54.26*	0.93 ± 0.08	1.72 ± 0.13
**PI 577630**	64.00*	0.80 ± 0.08	1.25 ± 0.12	**PI 660493**	55.05*	0.60 ± 0.19	1.09 ± 0.19
**PI 577633**	39.60*	0.84 ± 0.05	2.13 ± 0.07	**PI 660494**	13.51*	0.33 ± 0.21	2.47 ± 0.13
**PI 577634**	34.23*	0.76 ± 0.19	2.22 ± 0.58	**PI 660495**	50.85*	0.75 ± 0.06	1.47 ± 0.09
**PI 577636**	35.82*	0.80 ± 0.09	2.23 ± 0.16	**PI 660496**	39.06*	0.83 ± 0.06	2.13 ± 0.05
**PI 577637**	28.57*	0.40 ± 0.10	1.40 ± 0.4	**PI 660497**	28.49*	0.76 ± 0.10	2.66 ± 0.16
